# Effects of the Offset Term in Experimental Simulation on Afterglow Decay Curve

**DOI:** 10.1155/2014/497270

**Published:** 2014-06-26

**Authors:** Chi-Yang Tsai, Jeng-Wen Lin, Yih-Ping Huang, Yung-Chieh Huang

**Affiliations:** ^1^Graduate Institute of Dentistry, College of Oral Medicine, Taipei Medical University, Taipei 110, Taiwan; ^2^Department of Dentistry, Taipei Medical University Hospital, Taipei 110, Taiwan; ^3^Department of Civil Engineering, Feng Chia University, Taichung 407, Taiwan; ^4^Department of Pediatrics, Taichung Veterans General Hospital, Taichung 407, Taiwan

## Abstract

This study examines the effect of the offset term in a multiple single exponential equation that fits into experimental afterglow decay curve data for material applications. For afterglow materials applied and attached to structures, the inclusion of this offset term may reduce the values of the calculated decay times, *τ*_*i*_, and enlarge the time invariant constants, *A*_*i*_, in the associated equation compared to theoretically perfect test conditions. Using a set of experimental data obtained from a lab under dim light, adjustments can be made to calculate the required parameters for an equation without the offset term. This study uses mathematical simulations and lab tests to support our thesis and crosslink test results generated from different ambient light conditions. This paper defines the offset ratio as the ratio of the offset value, *I*_0_, versus the initial light intensity in an equation. This ratio can be used to evaluate possible effects on the calculated parameters of an equation in an associated numerical simulation. The most reliable parameters will have consistent results from the use of multiple single exponential equations, with and without the offset term, in simulations to obtain them in an equation to model a set of data.

## 1. Introduction

Many researchers commonly use empirical multiple single exponential equations to fit experimental curve data [1]. If a lab is unable to completely block ambient light or luminous intensity is not measured for a sufficient time period, residual light will appear in the test data, indicated by the offset term (*I*_0_) as follows:

(1) $$\begin{array}{c} I=I_{0}+\overset{n}{\underset{i=1}{\sum }}A_{i}\exp\left(\frac{-t}{\tau_{i}}\right), \end{array}$$

where *I* is the light intensity at any time, *t*, after switching off the excitation illumination, *I*_0_ is the offset term that represents residual light, and *A*_*i*_ is a time-invariant constant. The term *τ*_*i*_ is a decay constant (or decay time) for the exponential components. A choice can be made from among *n* = 1 as single, *n* = 2 as double, and *n* = 3 as triple exponential equations.

*I*_0_ was identified as background by Pedroza-Montero et al. and as background intensity by Chen et al. [2, 3]. In order to solve (1), the *I*_0_ term can be computed independently and subtracted from the *I* equation [4]. By quoting the physical properties of experimental data, Huang et al. showed methods to examine the definition for associated parameters in an equation and solve them [5]. Using log coordinates, Sharma et al. indicated that a decay curve, such as (1), can be treated as the minimum number of straight lines with slow, medium, and fast components, and each line represents a different light intensity stage [6]. In other words, *I*_0_ should not exist in good experimental data, and its influence on modeling must be investigated.

Theoretically, *I*_0_ should be zero in (1), and it is appropriate to use a zero value if the obscure light in the lab room is eliminated:

(2) $$\begin{array}{c} I=\overset{n}{\underset{i=1}{\sum }}A_{i}\exp\left(\frac{-t}{\tau_{i}}\right). \end{array}$$

The problem is that many researchers still use (1) for their studies [7–11]. This raises four questions.

1. Does the ambient light in a test affect the afterglow data locally or globally in a numerical simulation?
2. Can we adjust the experimental data received from a dim light environment to simulate that from a completely dark environment?
3. How does the difference in the calculated parameters used in (1) compare with (2)?
4. When can we stop an afterglow experimental test, even under perfect conditions?

This study focuses on answering the above questions using mathematical simulations and lab tests to support our results, that is, to find the effect of experimental data obtained from a dim light lab on the calculated parameters of an equation. Some dim light that exists in labs may be at an acceptable level, and the measured data can be adjusted for this using the conditions proposed in this paper. On the other hand, this may cause interpolation problems for experimental data obtained from a test that was not measured until the light intensity, *I*, was approximately zero.

## 2. Offset Ratio

Several lab tests were executed to study the effect of ambient light on the afterglow decay curve simulation.  Table 1 assigns the different conditions to the tests. Two kinds of patches were involved, PET resin and UV. The initial light intensity is expressed in (3), and the offset ratio (*R*) in (4) is defined as the ratio of the offset value (*I*_0_) versus the initial light intensity (*I*_initial_):

(3) $$\begin{array}{c} I_{\text{initial}}^{}=I_{0}+\overset{n}{\underset{i=1}{\sum }}A_{i}, \end{array}$$

(4) $$\begin{array}{c} R=\frac{I_{0}}{I_{\text{initial}}}, \end{array}$$

where *R* is the offset ratio in this paper, *I*_initial_ is the initial light intensity in a test, and *I*_0_ is the offset value of an equation.

**Figure 1:**
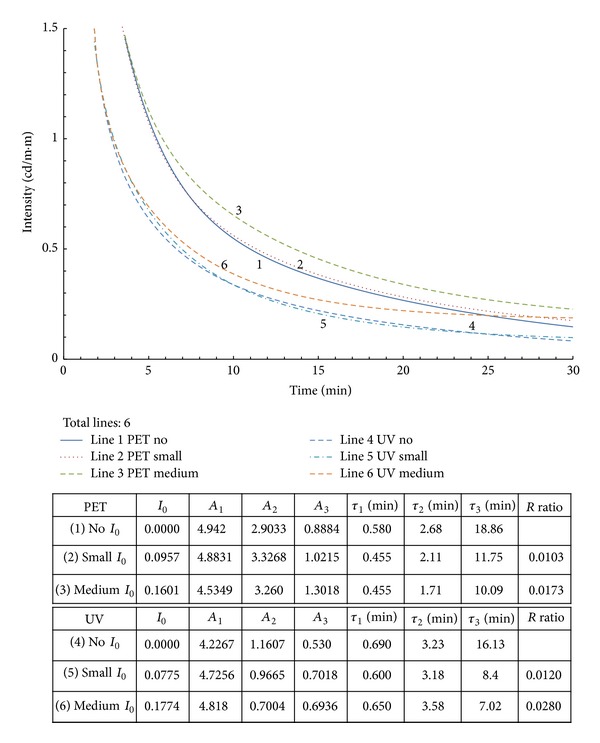
Decay curves used in this study as defined in Table 1.

It is obvious that with a larger offset ratio value (*R*), the processed curve (with *I*_0_) will greatly diverge from a perfect one (without *I*_0_).  Figure 1 depicts the decay curves and calculated parameters for all the cases described in Table 1. In this figure, there are three curves for PET (curves 1–3) and UV (curves 4–6) resins that are identical at the beginning. However, they gradually diverge at some point in time. Evidence shows that the ambient light only effects curves locally, but the diverge point is far away from the offset value, *I*_0_. The calculated parameters for these cases are tabulated on the lower side of the figure. They were calculated using triple exponential equations. Examination of the first three curves reveals that the largest is *τ*_3_ in curve 1 (no *A*_0_) calculated using (2). Curves 2-3 use (1) to compute the associated parameters. The results indicate that with an increase in the ambient light intensity, *τ*_3_ decreases with the increase of *A*_3_. The same behavior can be observed in curves 4–6. In that figure, curves 1 and 4 represent the perfect environmental testing, and the calculated parameters in the equation are regarded as correct. In other words, the cases performed with some ambient light will lead to an underestimation of the calculated decay times and an overestimation of the time-invariant constants. *τ*_3_ is usually taken to correlate to some phosphor behaviors [7–11].

## 3. The Quality of the Experimental Data

The quality of the experimental data is of vital importance for generating representative equations in curve simulations. In addition to accuracy in reading and machinery, there are several crucial processes that have to be dealt with, such as environmental and temperature controls, the time interval for data reading, and the duration of the test. Automatic reading by computer is required for such tests because the data can be measured within seconds. This also minimizes human error in reading the data. Another crucial issue is how much time is required for a good measurement. The next paragraph will focus on this subject.

Ideally, it is better to prolong a test as long as possible until the calibration limit is reached; however, this may mean that several hours are required for a single test run. The cost of an insufficient data duration for a numerical process is that variations in the equation interpolation will be part of the outcome. An easy way to identify this problem is to apply the two equations separately, with and without the offset term, such as with (1) and (2). Then, check to see if the corresponding parameters in the two equations have similar values. If they are in agreement with each other, then the data are appropriate for use. This may also mean the calculated parameters are acceptable.

Using some predefined parameters for a specific curve description in this study, we generated a set of data points that represent the curve before the separate application of (1) and (2) to compute the associated parameters in the equations. The data points are tabulated in Tables 2 and 3. If sufficient data are applied in the numerical interpolation, then, theoretically, regardless of the type of equation involved, a consistent result should be achieved. That is to say, the calculated parameters should be similar to the predefined parameters.

This study used data points that extend up to 120 min in the decay process and collected the calculated parameters in Tables 2 and 3. In both tables, the second row marked as “original” represents the predefined parameters in an afterglow equation and the third row marked as “120 min” shows the parameters calculated by including data points within a 120 min duration in the numerical process, and the same is true for “60 min” and other cases.

The data in Table 2 are calculated using an equation with the *I*_0_ term. It seems that 120 min is fine for the comparison with an opponent as without *I*_0_ case (Table 3) but with a slightly smaller *τ*_3_. Table 2 reveals that fewer data points in the interpolation can result in larger *I*_0_ values and small *τ*_3_ values. The last column is the offset ratio, *R*, and the *R* value is a direct response to the divergence of the calculated parameters. The smaller *R* value gives better results for interpolation. This study recommends that this value be kept below 1.0%, as seen in the example used here. Table 3 indicates that the calculated parameters are acceptable with inclusion of the data for more than 30 min. It is obvious that diverse results can be found in 9 min or less. Therefore, it is important to use the equation model without the *I*_0_ term for interpolation of an afterglow decay curve test.

## 4. Data Computational Adjustment

In Figure 1, curves 1 and 4 are used and the other curves resulted from imperfect testing conditions such as dim light. How can we transform curves 2-3 into curve 1 or 5 and curve 6 into curve 4?

**Figure 2:**
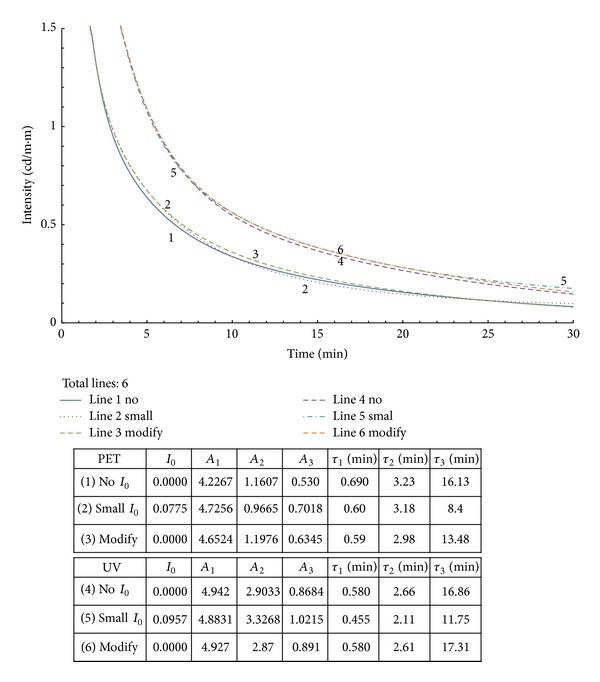
Modified curves with small *R* values from Figure 1.

**Figure 3:**
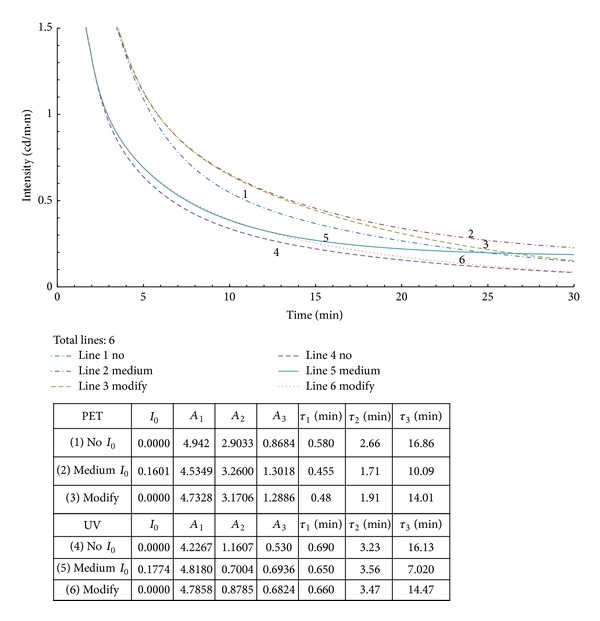
Modified curves with medium *R* values from Figure 1.

To revise the *τ*_*i*_ value calculated from (1), the lab data were adjusted by abandoning the last several terms that were smaller than the *I*_0_ term. Then, the data were reprocessed with (2). The results are plotted in Figures 2 and 3 for small and medium *R* values, respectively. The simulation had great results for the associated *τ*_3_ values. With larger *R* values, it is less likely that a good approximation can be achieved by merely trimming off the original data for the modification process as proposed. This topic deserves further study in the future.

## 5. Results and Discussion

**Figure 4:**
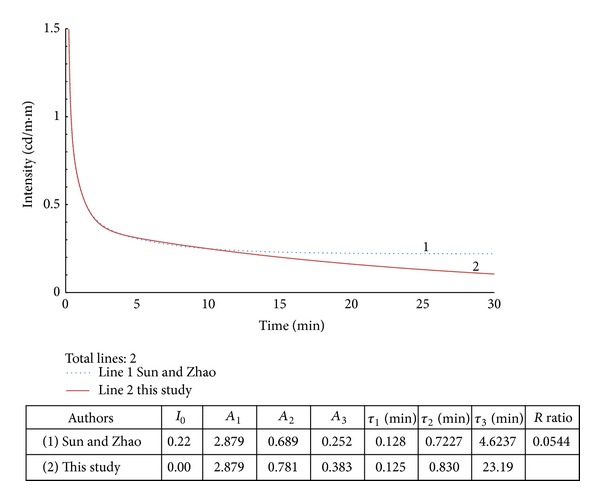
Example 1 with modified data from [7].

In this section, the proposed scheme discussed in the previous section is applied to the data reported in five different articles [7–11] for demonstration. We purposely chose cases processed with the offset term in a numerical approximation (1), and we recalculated for the parameters in (2) by removing the last several terms in the data. In other words, using the given parameters of an equation in previous articles, the experimental data along a decay curve can be recreated, and the last portion of the curve that is close or below the offset value is eliminated. The remaining data are used to generate a new curve from (2).  Figure 4 illustrates the first example. The calculated parameters are tabulated in the upper right hand side of the figure and include the original data as well as the data created for this study. The *R* value is 5.44%. This value is thought to be proportional to the difference between the two curves and their slow-decay times (*τ*_3_). The mismatch of 4.623 versus 23.19 for the two slow-decay times, *τ*_3_, in this example indicates that either the *R* value used is too large or (1) cannot be used to model this case.

**Figure 5:**
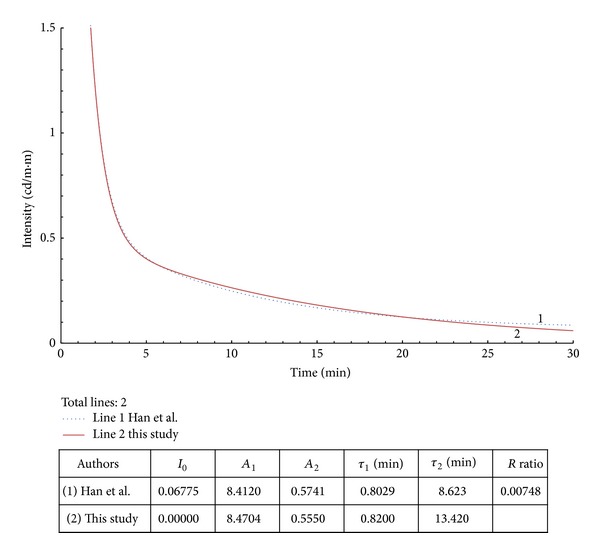
Example 2 with modified data from [8].

**Figure 6:**
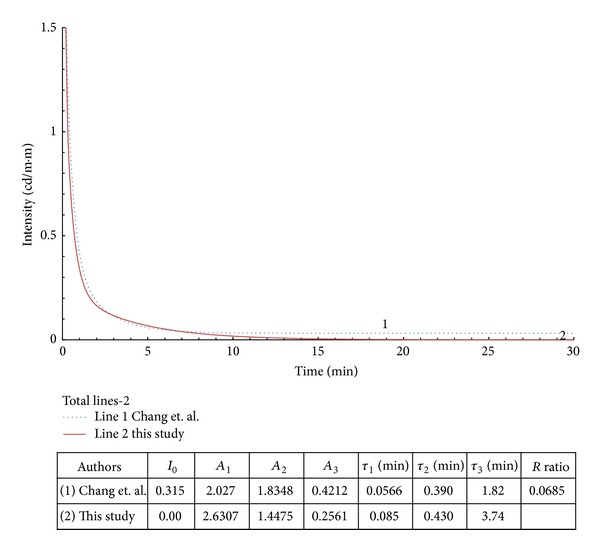
Example 3 with modified data from [9].

**Figure 7:**
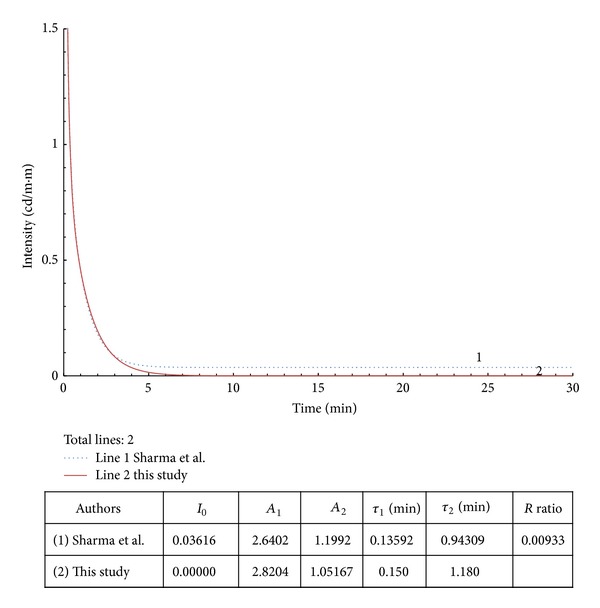
Example 4 with modified data from [11].

In examples 2–4 from Figures 5, 6, and 7, the *R* values are within 0.7%–6.8%. The regenerated curves and calculated parameters, *A*_*i*_ and *τ*_*i*_, are close to the original documented source. The results show that the calculated slow-decay times (*τ*_2_ or *τ*_3_) are approximately 2 times the original values.

**Figure 8:**
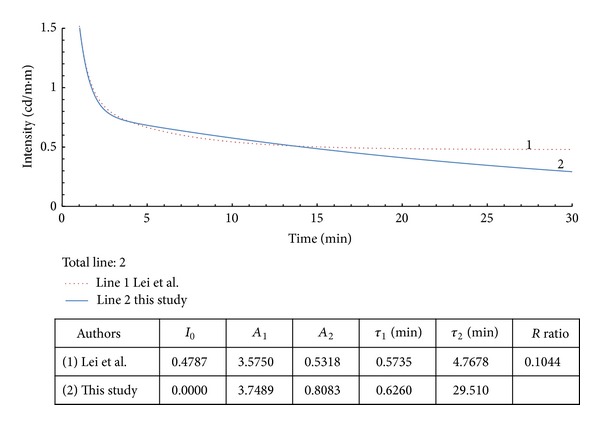
Example 5 with modified data from [10].

Example 5 shows an extreme situation with *R* values as high as 10.44%.  Figure 8 shows that the modified curve is close to the original one. However, the difference between the two slow-decay time values (*τ*_2_) in the table is 6 times greater. This shows that the experimental environment for afterglow luminescence measurements should be taken seriously.

## 6. Conclusions

It is common to retain dim light in afterglow experimental tests for different reasons, including easy operation, operator friendliness or ease of use, and physical chemistry applications of afterglow decay curve data. However, if such experimental data is directly used for numerical simulations, the consequence of having an offset term in the equation may cause confusion. The offset ratio is used to evaluate the possible effect of the offset term. This study points out that increasing the offset ratio may decrease the calculated slow-decay time (*τ*_2_ or *τ*_3_) from the use of (1) in interpolation, that is, mainly affecting the slow-decay time component in that equation. An adjustment scheme to modify the experimental data was proposed, and it was valid for compensating for the offset term effect in the equations. That is, a perfectly dark lab room may not be required to run the associated afterglow measurements. From the examples in this study, we recommend that the offset ratio should be less than 1.0% for such a test. The two equations, with and without offset terms, were used for a numerical process and then the generated parameters from these two equations were compared to examine the quality of the obtained experimental data.

## Figures and Tables

**Table 1:** The test conditions.

Cases	Resin	Initial intensity *I*_initial_ (cd/m^2^)	Ambient intensity *I*_0_ (cd/m^2^)	Offset ratio *R* = *I*_0_/*I*_initial_
(1) No *I*_0_	PET	9.200	0.000	0.000
(2) Small *I*_0_	PET	9.200	0.0957	0.010
(3) Medium *I*_0_	PET	9.200	0.1601	0.017
(4) No *I*_0_	UV	6.400	0.000	0.000
(5) Small *I*_0_	UV	6.400	0.0775	0.012
(6) Medium *I*_0_	UV	6.400	0.1774	0.028

**Table 2:** Simulated by using equations with the *I*_0_ term.

Source	*I*_0_	*A*_1_	*A*_2_	*A*_3_	τ_1_	τ_2_	τ_3_	*I*_0_/*I*_initial_
Original	0.0	4.90	2.90	0.87	0.60	2.70	16.90	
120 min	0.00223	4.9078	2.8833	0.878	0.60	2.70	16.55	0.026%
60 min	0.01327	4.7920	2.9271	0.942	0.59	2.54	15.14	0.153%
48 min	0.02047	4.7979	2.9127	0.945	0.59	2.54	14.74	0.236%
40 min	0.14750	3.2756	3.2200	2.049	0.50	1.24	6.17	1.697%
30 min	0.18213	3.5000	3.0774	1.942	0.50	1.34	6.06	2.093%

**Table 3:** Simulated by using equations without the *I*_0_ term.

Source	*I*_0_	*A*_1_	*A*_2_	*A*_3_	τ_1_	τ_2_	τ_3_
Original	0.00	4.90	2.90	0.87	0.60	2.70	16.90
120 min	0.00	4.8727	2.9230	0.8795	0.595	2.67	16.74
60 min	0.00	4.8727	2.9236	0.8795	0.595	2.67	16.74
30 min	0.00	4.8725	2.9239	0.8793	0.595	2.67	16.74
14 min	0.00	4.8875	2.8750	0.9250	0.600	2.66	16.08
9 min	0.00	4.8945	2.8388	0.9360	0.600	2.65	15.15
6 min	0.00	4.6317	2.2197	1.8210	0.585	1.91	7.59
